# Tocilizumab as a Potential Alternative Therapy for Acute Exacerbation of Systemic Sclerosis-Associated Interstitial Lung Disease in a Dialysis Patient Following Immunodeficiency-Associated Lymphoproliferative Disorders

**DOI:** 10.7759/cureus.105324

**Published:** 2026-03-16

**Authors:** Taiga Kuga, Makio Kusaoi, Ken Yamaji, Naoto Tamura

**Affiliations:** 1 Department of Internal Medicine and Rheumatology, Juntendo University School of Medicine, Tokyo, JPN

**Keywords:** connective tissue disease-associated interstitial lung disease, iatrogenic immunodeficiency-associated lymphoproliferative disorders, ssc-ild, systemic sclerosis, systemic sclerosis interstitial lung disease (ssc-ild), tocilizumab

## Abstract

A 62-year-old woman was diagnosed with diffuse cutaneous systemic sclerosis (dcSSc), complicated by scleroderma renal crisis, resulting in maintenance dialysis. Mycophenolate mofetil (MMF) and low-dose prednisolone stabilized her interstitial lung disease (ILD). Five years after the dcSSc diagnosis, she developed brain immunodeficiency‑associated lymphoproliferative disorders. Consequently, MMF was discontinued. Three months after MMF cessation, she developed a cough and productive sputum. Imaging revealed new ground-glass opacities (GGOs) in both lungs, indicating an acute exacerbation of systemic sclerosis-associated ILD (SSc-ILD). Tocilizumab was started, resulting in the rapid resolution of symptoms and new GGOs. Although this is a single case report, the findings suggest that tocilizumab may be a viable therapeutic option for SSc-ILD exacerbation, particularly in patients with limited treatment choices due to complex comorbidities.

## Introduction

Pulmonary involvement, primarily manifesting as interstitial lung disease (ILD) and pulmonary arterial hypertension, has been the leading cause of mortality in patients with systemic sclerosis (SSc)[1]. SSc-associated ILD (SSc-ILD) is characterized by progressive inflammation and fibrosis of the lung parenchyma, necessitating prompt and effective immunosuppressive therapy and anti-fibrotic drugs. According to the Scleroderma Lung Study II, mycophenolate mofetil (MMF) is considered a first-line treatment because it is more effective and safer than cyclophosphamide [2]. However, long-term immunosuppression carries the risk of rare but serious complications, such as immunodeficiency‑associated lymphoproliferative disorders (IA‑LPDs). 

IA-LPD comprises a broad spectrum of lymphoid proliferations arising in the setting of compromised immune function. According to the World Health Organization (WHO) classification, these disorders are categorized into four distinct groups based on the underlying cause: primary immune disorders, HIV infection, post-transplant settings (post-transplant lymphoproliferative disorders or PTLDs), and other iatrogenic factors (other iatrogenic immunodeficiency-associated lymphoproliferative disorders or OIIA-LPDs) [3]. OIIA-LPD occurs in patients receiving immunosuppressive therapy for autoimmune or inflammatory conditions and is considered a distinct clinical entity from PTLD. While most commonly associated with methotrexate, OIIA-LPD has also been reported with other agents, including MMF [4]. Although MMF is the first-line therapy for SSc-ILD [5], its long-term risk for IA-LPD remains unclear due to the scarcity of reported cases in the SSc population.

Recently, several studies have demonstrated that tocilizumab has a favorable effect in suppressing the progression of SSc-ILD [6,7] and is listed as a treatment option in the EULAR recommendations [5]. However, a significant knowledge gap exists regarding the optimal management of SSc-ILD exacerbation following the necessary discontinuation of MMF due to IA-LPD. In such cases, clinicians face a therapeutic dilemma: conventional rescue agents, such as rituximab or cyclophosphamide, are often avoided due to concerns over malignancy recurrence, while the underlying ILD requires active treatment. This case highlights the clinical utility of tocilizumab as a salvage therapy for the worsening of SSc-ILD in this challenging clinical scenario.

## Case presentation

Initial presentation and renal crisis

A 62-year-old woman was diagnosed with diffuse cutaneous systemic sclerosis five years before the current presentation, based on hand stiffness, skin thickening (modified Rodnan skin score or mRSS: 27), positive anti-Scl-70 antibodies, and presence of ILD. The following month, she was admitted due to a headache and hypertension of 185/89 mmHg. Laboratory findings revealed a significantly elevated serum creatinine level (6.44 mg/dL), anemia (hemoglobin: 9.6 g/dL and red blood cell count: 3.36 × 10¹²/L) with the presence of schistocytes, and thrombocytopenia (platelet count: 111 × 10⁹/L) (Table 1). These findings led to the diagnosis of scleroderma renal crisis (SRC) complicated by thrombotic microangiopathy. Despite treatment with captopril and plasma exchange, her renal function did not recover, and she commenced maintenance hemodialysis.

**Table 1:** Initial laboratory investigations and immunological findings ALT: alanine aminotransferase; ANA: antinuclear antibody; Anti-CCP antibody: anti-cyclic citrullinated peptide antibody; Anti-dsDNA antibody (RIA): anti-double-stranded DNA antibody (radioimmunoassay); Anti-SS-A antibody: anti-Sjögren's syndrome-related antigen A antibody; Anti-SS-B antibody: anti-Sjögren's syndrome-related antigen B antibody; Anti-U1-RNP antibody: anti-U1 ribonucleoprotein antibody; AST: aspartate aminotransferase; BUN: blood urea nitrogen; C3: complement component 3; C4: complement component 4; CH50: 50% hemolytic complement activity; CK: creatine kinase; Cre: serum creatinine; CRP: C-reactive protein; IgG/A/M: immunoglobulin G/A/M; KL-6: Krebs von den Lungen-6; LDH: lactate dehydrogenase; MPO-ANCA: myeloperoxidase-antineutrophil cytoplasmic antibody; PR3-ANCA: proteinase 3-antineutrophil cytoplasmic antibody; SP-D: surfactant protein D; γ-GT: gamma-glutamyl transferase.

Parameter	Value	Unit	Reference range
White blood cell count	8800	/μL	3600-8900
Red blood cell count	3.36	x 10^12^/L	3.80- 5.04
Hemoglobin	9.0	g/dL	11.1-15.2
Platelet count	111	x 10^9^/L	153-346
Erythrocyte sedimentation rate	105	mm/h	0-20
AST	113	U/L	5-37
ALT	26	U/L	6-43
LDH	1426	U/L	119-221
γ-GT	17	U/L	0-75
CK	468	U/L	47-200
BUN	98	mg/dL	9-21
Cre	6.44	mg/dL	0.50-0.80
CRP	0.43	mg/dL	0-0.29
KL-6	698	U/mL	0-499
SP-D	194	ng/mL	0-109.9
IgG	1516	mg/dL	870-1700
IgA	243	mg/dL	110-410
IgM	58	mg/dL	46-260
C3	123	mg/dL	69-128
C4	31	mg/dL	14-36
CH50	73.8	U/mL	25-54
Rheumatoid factor	14.1	IU/mL	0-15
Anti-CCP antibody	<0.6	U/mL	0-4.4
Antinuclear antibody	160	-	0-19
ANA pattern	Homogeneous nucleolar speckled	-	-
Anti-U1-RNP antibody	Negative	-	Negative
Anti-SS-A antibody	Negative	-	Negative
Anti-SS-B antibody	Negative	-	Negative
Anti-dsDNA antibody (RIA)	<2.0	IU/mL	0-6
Anti-Scl-70 antibody	8	-	Negative
Anti-centromere antibody	Negative	-	Negative
Anti-RNA polymerase III antibody	Negative	-	Negative
MPO-ANCA	<1.0	U/mL	0-3.4
PR3-ANCA	<1.0	U/mL	0-3.4

Management of ILD and emergence of IA‑LPD

**Figure 1:**
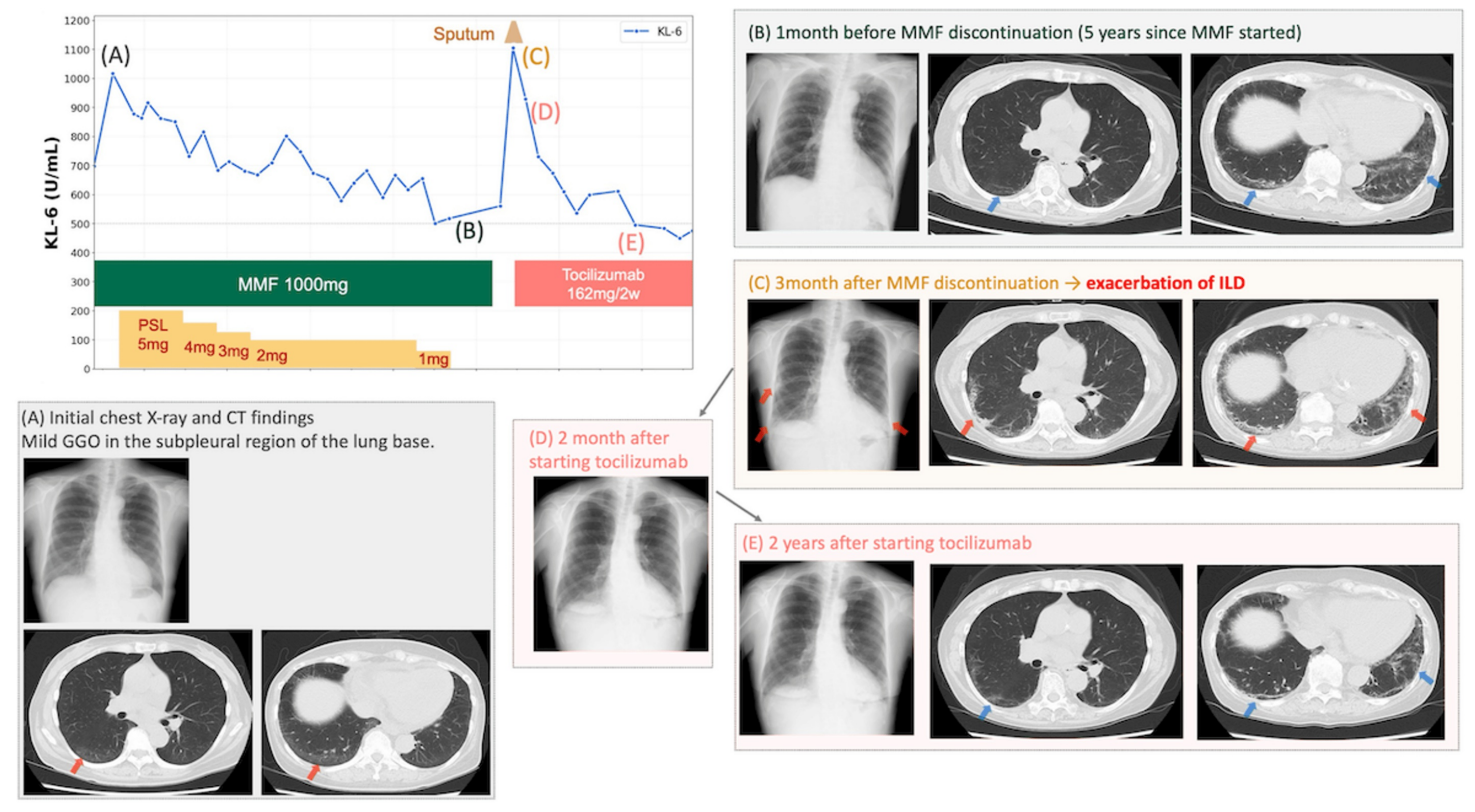
Clinical course, serum KL-6 levels, and longitudinal imaging findings The graph illustrates the clinical course, plotting serum KL-6 levels (U/mL) on the y-axis and time on the x-axis. Color bars indicate the duration of treatment with mycophenolate mofetil (MMF), prednisolone (PSL), and tocilizumab. The labels (A)–(E) on the timeline correspond to the chest imaging findings shown in the panels. Radiographic findings at each indicated time point: (A) Baseline (at initial diagnosis): Chest radiography and CT scan reveal mild subpleural ground-glass opacities (GGOs) in the bilateral lung bases. (B) Pre-discontinuation (one month before MMF cessation): Stable interstitial lung disease (ILD) findings after five years of MMF therapy. (C) Exacerbation (three months post-MMF discontinuation): Marked relapse of ILD, characterized by the emergence of new and worsening peripheral GGOs and increased sputum production. (D) Early response (one month after tocilizumab initiation): Follow-up X-ray shows early radiographic improvement. (E) Long-term resolution (18 months after tocilizumab initiation): Subsequent CT scan confirms near-complete resolution of new GGOs, correlating with the normalization of serum KL-6 levels.

**Figure 2:**
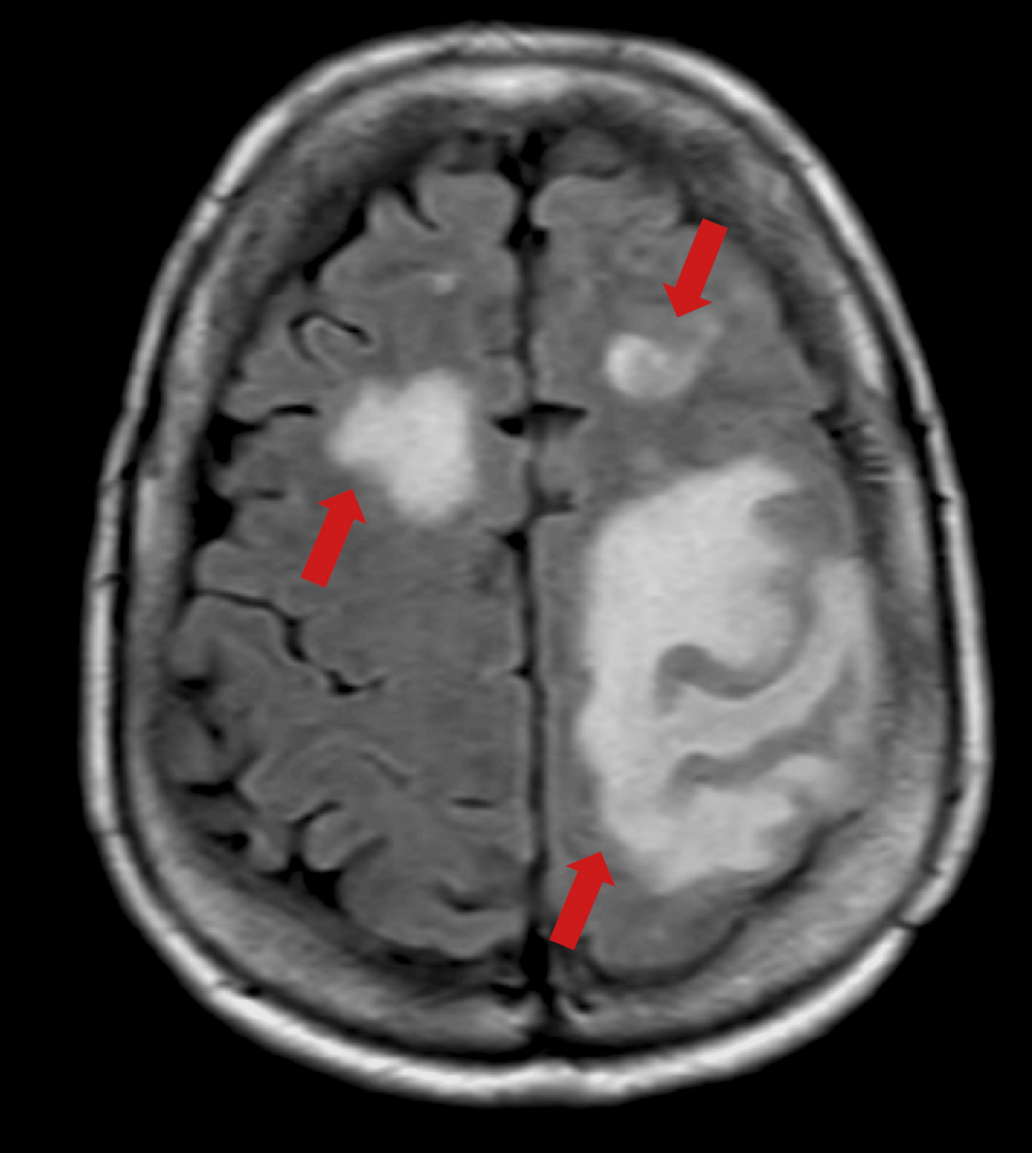
Brain MRI findings of intracranial IA-LPD Fluid-attenuated inversion recovery (FLAIR) MRI reveals patchy hyperintense signals involving the right frontal and left temporal lobes, extending toward the frontal and parietal regions. IA-LPD: immunodeficiency‑associated lymphoproliferative disorder.

Baseline chest computed tomography (CT) revealed subpleural ground-glass opacities (GGOs) in both lung bases, and laboratory findings showed elevated serum levels of ILD markers, including Krebs von den Lungen-6 (KL-6) (698 U/mL) and surfactant protein D (194 ng/mL). Pulmonary function tests (PFTs) demonstrated a restrictive respiratory defect: forced vital capacity (FVC) of 1.99 L (78.7% predicted), forced expiratory volume in 1 second (FEV1) of 1.70 L (84.2% predicted), and diffusing capacity of the lung for carbon monoxide (DLCO) of 11.40 mL/min/mmHg (54.9% predicted). Treatment with MMF was initiated at a dose of 1000 mg/day two months after diagnosis (Figure 1). After three months, serum KL-6 levels increased to 1016 U/mL, and 5 mg/day of prednisolone (PSL) was carefully initiated. Serum KL-6 levels were decreased to around 500-600 U/mL, and PSL was tapered and then discontinued (Figure 1). Four years and nine months after diagnosis, she developed a tremor in her right hand and eyelid twitching. A brain MRI identified a mass in the right frontal and left temporal lobes (Figure 2). A craniotomy and brain biopsy performed two months later confirmed a diagnosis of IA‑LPD (lymphomatoid granulomatosis, grade 3, with the presence of Epstein-Barr virus-positive large B cells). Consequently, MMF was discontinued in the following month. After discontinuing MMF, the brain tumor showed spontaneous shrinkage, and her neurological symptoms improved.

Progression of ILD and response to tocilizumab

Three months after stopping MMF, the patient developed a persistent cough and increased sputum production. A physical examination revealed fine crackles at the lung bases, and her peripheral oxygen saturation or SpO_2_ was 97% (room air). Laboratory tests showed an elevated lactate dehydrogenase (579 U/L), KL-6 (1105 U/mL), and CRP (6.91 mg/dL) (Table 2).

**Table 2:** Laboratory findings at the time of interstitial lung disease progression ALT: alanine aminotransferase; AST: aspartate aminotransferase; BUN: blood urea nitrogen; Cre: serum creatinine; CRP: C-reactive protein; LDH: lactate dehydrogenase; KL-6: Krebs von den Lungen-6; γ-GT: gamma-glutamyl transferase.

Parameter	Value	Unit	Reference range
White blood cell count	6100	/μL	3600-8900
Red blood cell count	4.30	x 10^12^/L	3.80-5.04
Hemoglobin	11.4	g/dL	11.1-15.2
Platelet count	240	x 10^9^/L	153-346
Erythrocyte sedimentation rate	100	mm/h	0-20
AST	59	U/L	5-37
ALT	8	U/L	6-43
LDH	579	U/L	119-221
γ-GT	19	U/L	0-75
BUN	42	mg/dL	9-21
Cre	4.78	mg/dL	0.50-0.80
CRP	6.91	mg/dL	0-0.29
KL-6	1105	U/mL	0-499

A chest CT scan revealed new bilateral GGOs, primarily in the lower lobes. Although a sputum culture was not obtained, the patient had already tested negative for both influenza and SARS-CoV-2 via rapid antigen tests at a local clinic. Given the negative blood tests (including T-SPOT.TB and β-D-glucan) and the absence of fever, the clinical likelihood of infection was deemed low. Furthermore, volume overload was excluded due to stable dialysis control without cardiomegaly or pleural effusion, and paraneoplastic changes were improbable, as the intracranial IA-LPD lesion had already demonstrated regression following MMF withdrawal. Although a lung biopsy was not feasible to distinguish among inflammatory pneumonitis, secondary organizing pneumonia, and infection, the acute onset of subpleural GGOs and elevated CRP and KL-6 was highly suggestive of an acute exacerbation of SSc-ILD. Given the history of IA-LPD, re-administration of MMF or initiation of other potent immunosuppressants, including rituximab or high-dose corticosteroids, was deemed high risk due to the potential for malignancy recurrence. Furthermore, nintedanib was not selected because acute-onset, GGO-dominant lesions were suggestive of active inflammation rather than chronic progressive fibrosis. In this clinical dilemma, we chose tocilizumab as a targeted alternative to mitigate the inflammatory process while minimizing generalized immunosuppression, particularly by avoiding agents that might hinder ongoing immune recovery from IA-LPD.

Notably, the patient’s respiratory symptoms resolved rapidly within two weeks. Follow-up chest radiography at two months confirmed significant improvement in the newly developed opacities in the right middle lung field and bilateral lung bases. Subsequent chest CT scans further confirmed the nearly complete resolution of the new GGOs. Consistent with the radiographic findings, follow-up PFTs demonstrated stabilization of lung function. Compared to the baseline values at diagnosis, DLCO remained largely stable (from 11.40 to 12.32 mL/min/mmHg; 60.7% predicted), as did FVC (1.91 L; 82.3% predicted) and FEV1 (1.65 L; 89.1% predicted). These results indicate that pulmonary function was well preserved following initiation of tocilizumab, without further deterioration despite the prior acute flare. Furthermore, serum KL-6 levels normalized to 449 U/mL (within the normal range), further confirming the favorable clinical response, including stable skin fibrosis (mRSS: 26). These results indicate that the treatment effectively reversed the acute flare and prevented long-term functional decline. During the 30-month follow-up period after MMF withdrawal, the patient remained free of IA-LPD recurrence while receiving tocilizumab monotherapy. While this clinical course is encouraging, the long-term impact of tocilizumab on the risk of IA-LPD recurrence remains unknown and requires further accumulation of cases.

## Discussion

The management of SSc-ILD exacerbation in this patient presented a profound therapeutic dilemma. First, the patient’s history of SRC restricted the use of high-dose corticosteroid therapy. In SSc, especially the diffuse cutaneous subtype, prednisolone doses greater than 15 mg/day are well-recognized risk factors for triggering or exacerbating SRC [5]. Since the patient was already undergoing maintenance hemodialysis following a previous SRC event, aggressive steroid use to control the ILD flare was deemed too risky. Second, cyclophosphamide was not a viable option. In patients on hemodialysis, delayed clearance of cyclophosphamide metabolites significantly increases the risk of severe and prolonged bone marrow suppression. Furthermore, the use of potent cytotoxic agents such as cyclophosphamide was particularly avoided. While IL-6 blockade could theoretically impair immune surveillance against EBV, broad cytotoxic agents may carry a risk of severe, generalized immunosuppression that may more profoundly hinder the immune reconstitution necessary for spontaneous LPD regression. In methotrexate-associated LPD cases, the median time to maximal effect after withdrawing immunosuppressants is 12 weeks, with some cases taking significantly longer [8]. At the time of the ILD flare (12 weeks post-withdrawal), the patient’s neurological symptoms persisted, and complete radiological resolution was not yet achieved. In this delicate phase, we prioritized avoiding the T-cell toxicity associated with cyclophosphamide. However, the comparative safety of IL-6 inhibitors vs. cytotoxic agents in IA-LPD remains unproven, and this single case does not establish the oncologic safety of tocilizumab.

In this clinical dilemma, we sought clinical justification from existing safety data in other conditions. Long-term surveillance data from rheumatoid arthritis (RA) patients have not shown a significantly increased risk of malignancy associated with tocilizumab [9]. Furthermore, recent evidence suggests that tocilizumab does not increase the risk of cancer recurrence in patients with RA and a history of solid cancer before remission [10]. While these findings from the RA population cannot be directly extrapolated to patients with recent IA-LPD, they provided a cautious rationale for using tocilizumab as a potentially less cytotoxic alternative. In light of these considerations and its emerging role in SSc-ILD, we opted for tocilizumab, which is FDA-approved for SSc-ILD and endorsed by EULAR recommendations [5], although it remains an off-label treatment for this specific indication in Japan.

Given the associated risks, we provided the patient with a thorough and dedicated explanation of the treatment's benefits and potential complications. Informed consent was obtained, and the treatment was initiated based on the patient's autonomous and voluntary decision. It has been reported that physicians and patients often hold divergent cognitive models regarding SSc-ILD, which can create significant communication challenges; thus, enhancing the SDM process is essential for navigating complex treatment landscapes [11].

The rapid clinical response in this case is likely due to the inflammatory nature of SSc-ILD progression. IL-6 is a critical mediator in SSc pathogenesis [12], and elevated circulating levels are known to identify patients at high risk of progressive lung fibrosis [13]. The 2023 EULAR recommendations suggest considering tocilizumab for "early" SSc-ILD, according to the inclusion criteria of the focuSSced trial, which was within 60 months of disease onset [7]. In our case, we initiated tocilizumab 63 months from onset. Although this slightly exceeded the trial's criteria, it was considered clinically consistent with the "early" phase. Notably, while the focuSSced trial established tocilizumab’s role in preserving lung function by slowing FVC decline (-0.4% vs. -4.6% at 48 weeks) [7], our case extends this role by demonstrating its efficacy in reversing active inflammatory changes, going beyond merely preventing disease progression. Further reports and large-scale analyses are needed to refine patient selection through a more phenotype-driven approach.

There are several limitations to this report. First, the radiological assessment was based on qualitative clinical interpretation rather than a standardized scoring system or quantitative CT analysis. Furthermore, the diagnosis lacked histopathologic confirmation through lung biopsy, and we could not rigorously exclude other differential diagnoses, such as organizing pneumonia or occult infection, which can mimic the radiological patterns of SSc-ILD. However, the temporal correlation between MMF withdrawal, the clinical flare, and the subsequent response to tocilizumab was sufficiently robust to support our clinical conclusions. Second, we did not measure serum IL-6 levels or longitudinal trends in inflammatory biomarkers, which limits our ability to provide direct pathophysiological evidence for the observed clinical response. While the impact of tocilizumab on such immune recovery remains not fully understood, we prioritized it as a potentially less T-cell-toxic alternative to conventional cytotoxic therapy in this specific clinical dilemma. However, it must be emphasized that the absence of IA-LPD recurrence in this single case does not establish the long-term oncologic safety of tocilizumab, and its use in patients with a history of IA-LPD requires careful monitoring.

## Conclusions

This case supports further investigation regarding the possible efficacy of tocilizumab in treating acute exacerbations of SSc-ILD, particularly in patients with serious complications such as a history of SRC, dialysis, or malignancy. Although limited by its single-case nature, the lack of histopathologic confirmation, the absence of direct IL-6 data, and the potential for overlapping clinical phenotypes such as organizing pneumonia, the rapid response observed in this case supports the consideration of tocilizumab as a possible alternative therapy for acute exacerbations of SSc-ILD with a likely inflammatory phenotype.

## Appendices

We obtained informed consent for the publication of clinical data and images. A detailed record of the informed consent was maintained within the patient's medical files.
